# Pieces in a global puzzle: Population genetics at two whale shark aggregations in the western Indian Ocean

**DOI:** 10.1002/ece3.8492

**Published:** 2022-01-25

**Authors:** Royale S. Hardenstine, Song He, Jesse E. M. Cochran, Camrin D. Braun, Edgar Fernando Cagua, Simon J. Pierce, Clare E. M. Prebble, Christoph A. Rohner, Pablo Saenz‐Angudelo, Tane H. Sinclair‐Taylor, Gregory B. Skomal, Simon R. Thorrold, Alexandra M. Watts, Casey J. Zakroff, Michael L. Berumen

**Affiliations:** ^1^ Division of Biological and Environmental Science and Engineering Red Sea Research Center King Abdullah University of Science and Technology Thuwal Saudi Arabia; ^2^ Biology Department Woods Hole Oceanographic Institution Woods Hole Massachusetts USA; ^3^ School of Biological Sciences Centre for Integrative Ecology University of Canterbury Christchurch New Zealand; ^4^ WorldFish Bayan Lepas Malaysia; ^5^ Marine Megafauna Foundation Truckee California USA; ^6^ National Oceanography Centre University of South Hampton South Hamton UK; ^7^ Facultad de Ciencias Instituo de Ciencias Ambientales y Evolutivas Universidad Austral de Chile Valdivia Chile; ^8^ Australian Institute of Marine Science Townsville Qld Australia; ^9^ Massachusetts Division of Marine Fisheries New Bedford Massachusetts USA; ^10^ Ecological Genetics and Conservation Laboratory Manchester Metropolitan University Manchester UK

**Keywords:** genetic diversity, global population structure, microsatellites, mtDNA, *Rhincodon typus*

## Abstract

The whale shark *Rhincodon typus* is found throughout the world's tropical and warm‐temperate ocean basins. Despite their broad physical distribution, research on the species has been concentrated at a few aggregation sites. Comparing DNA sequences from sharks at different sites can provide a demographically neutral understanding of the whale shark's global ecology. Here, we created genetic profiles for 84 whale sharks from the Saudi Arabian Red Sea and 72 individuals from the coast of Tanzania using a combination of microsatellite and mitochondrial sequences. These two sites, separated by approximately 4500 km (shortest over‐water distance), exhibit markedly different population demographics and behavioral ecologies. Eleven microsatellite DNA markers revealed that the two aggregation sites have similar levels of allelic richness and appear to be derived from the same source population. We sequenced the mitochondrial control region to produce multiple global haplotype networks (based on different alignment methodologies) that were broadly similar to each other in terms of population structure but suggested different demographic histories. Data from both microsatellite and mitochondrial markers demonstrated the stability of genetic diversity within the Saudi Arabian aggregation site throughout the sampling period. These results contrast previously measured declines in diversity at Ningaloo Reef, Western Australia. Mapping the geographic distribution of whale shark lineages provides insight into the species’ connectivity and can be used to direct management efforts at both local and global scales. Similarly, understanding historical fluctuations in whale shark abundance provides a baseline by which to assess current trends. Continued development of new sequencing methods and the incorporation of genomic data could lead to considerable advances in the scientific understanding of whale shark population ecology and corresponding improvements to conservation policy.

## Graphical abstract

This study adds samples from whale sharks in two understudied regions, describes new microsatellite markers, proposes an incremental improvement to mitochondrial analysis, and provides a point of comparison to previous results from other aggregations. We include the first genetic sequences for whale sharks from the Mafia Island, Tanzania aggregation, and nearly quadruple those available from the Saudi Arabian Red Sea. These sequences were analyzed alongside 699 publicly available sequences from other aggregations. Our results demonstrate that our two study aggregations are derived from the same source population, despite strong ecological differences shown in previous work.

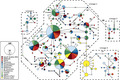

## 1 INTRODUCTION

The whale shark, *Rhincodon typus* Smith 1828, has often been described as an enigmatic species (Rowat & Brooks, 2012). Despite its size (up to 20 m) (Chen et al., 1997) and circumglobal distribution, encounters with these ocean giants were once rare, with only 320 records documented between 1801 to 1985 (Wolfson & Sciara, 1981). Smith's initial description of the species and virtually all early whale shark research was based on chance encounters. The discovery of sites with predictable whale shark aggregations resulted in a rapid increase in data available for the species (Norman et al., 2017). Unfortunately, these data have shown sustained population declines, leading the International Union for Conservation of Natural Resources (IUCN) Red List to change the species’ global status from Vulnerable (Norman, 2005) to Endangered (Pierce & Norman, 2016). An improved understanding of the whale shark's global and regional population structure and the species’ patterns of dispersal can help determine the scale of management units. This is critical to long‐term conservation efforts and planning because management strategies for a collection of independent aggregation sites may not be effective when applied to a wide‐ranging species that exhibits functional connectivity across basins.

Most whale shark population and movement ecology studies are based on photo‐identification (Araujo et al., 2014; Cochran et al., 2016; McKinney et al., 2017; Norman, Holmberg, et al., 2017; Robinson et al., 2016; Rohner et al., 2015) and/or telemetry (Berumen et al., 2014; Cagua et al., 2015; Cochran et al., 2019; Hueter et al., 2013; Norman, Whitty, et al., 2017; Robinson et al., 2016). While these methods have been successfully used to describe local population structure and to track movements within, between, and away from aggregation sites, they are limited by their focus on known aggregations (Sequeira et al., 2012). Most of these sites are dominated by either subadult males (Araujo et al., 2014; Diamant et al., 2018; Graham & Roberts, 2007; Ketchum et al., 2013; McKinney et al., 2017; Meekan et al., 2006; Ramírez‐Macías et al., 2012; Riley et al., 2010; Robinson et al., 2016; Rohner et al., 2015; Rowat et al., 2011), or more rarely, mature females (Acuña‐Marrero et al., 2014; Ketchum et al., 2013). Both cases imply that much of the current data are demographically skewed. Mature sharks (particularly males), subadult females, and neonates of both sexes are underrepresented (Norman, Holmberg, et al., 2017). Genetic comparison among aggregations can overcome this issue because patterns in molecular data reflect the distribution and movement of previous generations, as well as those of sampled individuals, effectively providing a more holistic view of the species’ demography across an evolutionary timescale.

Three independent global investigations of whale shark population genetics have been conducted: one using the mitochondrial control region (Castro et al., 2007), one using microsatellite loci (Schmidt et al., 2009), and one using both marker types (Vignaud et al., 2014). These studies show that genetic differentiation exists between whale sharks from the Indo‐Pacific and Atlantic Oceans, and additional samples from more recent research (Meekan et al., 2017; Sigsgaard et al., 2017; Yagishita et al., 2020) have largely corroborated this result. The Indo‐Pacific and Atlantic regions were subsequently treated as large‐scale genetic “subpopulations” for the IUCN global assessment (Pierce & Norman, 2016). In addition to the delineation of basin‐scale populations, one of the global comparison studies demonstrated a 6‐year decline in genetic diversity of whale sharks that were sampled from Western Australia (Vignaud et al., 2014). However, the same study also found evidence of a recent population expansion within the broader Indo‐Pacific (Vignaud et al., 2014), which has been confirmed by more recent work (Yagishita et al., 2020). Regardless, local assessments of temporal trends in genetic diversity have not been conducted at any aggregation sites outside of Western Australia.

Limited connectivity has been observed among the whale shark aggregation sites identified within the broader western Indian Ocean region. Photo‐identification (Andrzejaczek et al., 2016; Brooks et al., 2010; Norman, Holmberg, et al., 2017; Robinson et al., 2016), telemetry (Berumen et al., 2014; Brunnschweiler et al., 2009; Cagua et al., 2015; Norman, Whitty, et al., 2017; Robinson et al., 2016; Rohner et al., 2020), and biochemical (Prebble et al., 2018) studies have not shown any direct evidence of large‐scale connectivity between distant sites within this basin. Here, whale shark tissue samples collected from aggregation sites in the Saudi Arabian Red Sea (Berumen et al., 2014) and Mafia Island, Tanzania (Cagua et al., 2015) were analyzed. Genetic similarity between the two sites was assessed using both microsatellite markers (including novel primers developed for this study) and the whale shark mitochondrial control region. The same markers were also used to track genetic diversity within the Saudi Arabian Red Sea, providing the first direct comparison to similar studies from Western Australia (Vignaud et al., 2014). The novel sequences from both Saudi Arabia and Tanzania were combined with previously published data and used to generate updated global haplotype networks. These networks map the genetic similarity of sampled sharks based on mitochondrial sequences and can be used to identify geographic patterns in the molecular data.

## 2 MATERIAL AND METHODS

### 2.1 Ethics statement

This research was undertaken in accordance with the policies and procedures of the King Abdullah University of Science and Technology (KAUST) and approved by KAUST’s Institutional Biosafety and Bioethics Committee and Institutional Animal Care and Use Committee (protocol #20IACUC07). Permissions relevant for KAUST to undertake research have been obtained from the applicable governmental agencies in the Kingdom of Saudi Arabia. All works in Tanzania were conducted with the approval from the Tanzanian Commission for Science and Technology (COSTECH #2018‐545‐NA‐2015‐161).

### 2.2 Field sites

**FIGURE 1:**
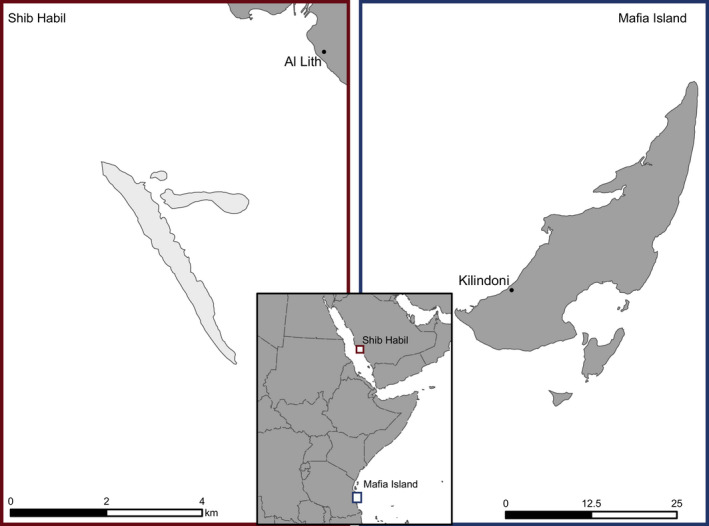
Two main sampling locations for this study. Center: Both locations shown in regional context. Shib Habil, Saudi Arabia, highlighted in red box and expanded to the left. Mafia Island, Tanzania, highlighted in blue box expanded on the right. Maps were composed in ArcPro (Esri Inc.) using layers sourced from MF Campbell Jr

Shib Habil is a submerged reef platform in the Red Sea approximately 5 km from the Saudi Arabian port city of Al Lith (Figure 1). This site attracts juvenile whale sharks of both sexes, and shark presence is highly seasonal. Most whale shark encounters at Shib Habil occur in March, April, or May (Cochran et al., 2016), after which the animals disperse into the wider Red Sea and Indian Ocean (Berumen et al., 2014). The Tanzanian aggregation site is located in Kilindoni Bay to the southwest of Mafia Island (Figure 1). Here, most of the sharks are juvenile males, and are commonly seen feeding on sergestid shrimp from November through February with sightings declining through March (Rohner, Richardson, et al., 2015; Rohner, Armstrong, et al., 2015). Acoustic telemetry has revealed that many of the sharks remain cryptically present within Kilindoni Bay or close by year‐round, with two to four consecutive years of high residence recorded for some individuals (Cagua et al., 2015; Rohner et al., 2020).

### 2.3 Sample collection

Whale shark tissue was collected over six seasons in Saudi Arabia (2010–2015) and over two seasons in Tanzania (late 2012 to early 2014). Sampling at both sites occurred during their respective whale shark tourism seasons and followed the same procedure. Free‐swimming whale sharks were approached by snorkelers and tissue samples were taken using a Hawaiian sling pole‐spear fitted with a biopsy tip. When possible, researchers estimated size, determined sex, and collected photos for individual identification. Identification photos were analyzed using both the Groth (Arzoumanian et al., 2005) and the Interactive Individual Identification System (I^3^S) (Van Tienhoven et al., 2007) algorithms to identify individuals and flag potential duplicate samples (Cochran et al., 2016; Rohner, Richardson, et al., 2015). Suspected duplicates were retained and eventually sequenced to confirm photo identification and to ensure that the highest quality sample was used for further analysis. Collected samples were preserved immediately in 70%–90% ethanol, or, in cases where ethanol was not available, samples were put on ice and transferred to a freezer. All samples were eventually placed in 90% ethanol and kept at −20℃ for long‐term storage.

### 2.4 DNA extraction and PCR

Each sample consisted of white, subcutaneous tissue and a black dermal‐cap. The dermal‐cap was separated with a scalpel and used for all further analyses. DNA was extracted using one of two kits, the DNeasy Blood and Tissue Kit (Qiagen Inc.) or the NucleoSpin Tissue Kit (Macherey‐Nagel), following the respective kit instructions. The quality and quantity of extracted DNA were measured using a NanoDrop 8000 spectrophotometer (Thermo Scientific; www.thermoscientific.com). Next‐generation sequencing was performed for one specimen using a Roche 454 GS FLX (titanium) sequencer and genomic library was constructed following the manufacturer's protocol. Raw unassembled reads from this library were mined for putative microsatellite loci using the msat‐commander v 1.0.8 software (Faircloth, 2008). Default settings were used to screen perfect dinucleotide and tetranucleotide repeats that were at least 20 bp long, resulting in 1588 putative microsatellite loci. Primer 3 (Rosen & Skaletsky, 2000) software was then used to design 353 primer pairs for all reads that contained suitable microsatellite repeat motifs. From these 14 novel primer pairs were selected and synthesized along with 8 primers from earlier publications (three from Ramirez‐Macias et al., 2009, five from Schmidt et al., 2009), producing 22 candidates for PCR trials. (Appendix S1: SI 1) PCR reactions were run with 5 µl of Master Mix (Qiagen Inc.), 3 µl of RNA free water, 1 µl of primer mix (each primer concentration 1 µM), and 1 µl of template DNA. The PCR reactions were initiated at 95℃ for 15 min, followed by 35 cycles of 94℃ for 30 s, 55℃ for 90 s, and 72℃ for 60 s, and concluded at 60℃ for 30 min. PCR products were then diluted to 1:50 with water before being sent for fragment analysis.

For mitochondrial analysis, the whale shark control region (primers WSCR1‐F and WSCR2‐R) from Castro et al. (2007) was chosen for comparison with previous studies and publicly available sequences. A 12.5 µl solution of Master Mix (Qiagen Inc.) (6.25 µl), water (4.25 µl), forward and reverse primers (0.5 µl each at 10 µM concentration), and template DNA (1 µl) was added to each well. The thermocycler program was as follows: 95℃ for 15 min; 35 cycles of 94℃ for 60 s, 58℃ for 60 s, and 72℃ for 45 s; and a final elongation step at 72℃ for 10 min. A subsample of PCR product was checked using Qiaxcel (Qiagen Inc.). Following this, PCR products were cleaned using ExoSap‐IT (Affymetrix). Purified products were sequenced using an ABI 3730xl platform.

### 2.5 Microsatellite marker analyses

Microsatellite markers were used to assess local patterns in genetic diversity within and between the Tanzanian and Saudi Arabian aggregations. These markers are derived from nuclear DNA, so they reflect both the maternal and paternal histories of the sampled animals. Microsatellite allele size was read using Geneious 8.1.6 software (Biomatters Ltd.). After scoring, any duplicate genotypes were identified using the Microsatellite Toolkit (Park, 2001). A subset of duplicate sequences was then used to calculate genotyping error before being removed from further analysis. Primers were screened using Genepop 4.2 (Rousset, 2008) to exclude markers containing either null alleles or linked loci and to ensure Hardy–Weinburg equilibrium. The remaining loci were used for all further microsatellite analyses. In order to assess genetic diversity within the two aggregations, each site's expected and observed heterozygosity were compared using GenAlEx version 6.4 (Peakall & Smouse, 2006) and local genetic diversity was determined using the Allelic Diversity Analyzer (ADZE) version 1.0 (Szpiech et al., 2008) via the rarefaction method. This technique analyzes private alleles and allelic richness within populations and is designed to be robust when sampling sizes vary. ADZE was used to determine overall genetic diversity for both the Tanzanian and Saudi Arabian populations individually. Genetic diversity was also calculated independently for each of the Saudi Arabian aggregation's five sampling seasons and compared using a Mann–Kendall trend test (Kendall, 1955; Mann, 1945) to determine if any substantial changes in local diversity occurred from 2010 to 2015.

Differentiation between the two aggregations was quantitated using a pairwise fixation index (*F*_ST_) and Shannon's mutual information, both of which were calculated using on GenAlEx version 6.4 (Peakall & Smouse, 2006). The same program was also used to identify any alleles found at only one of the two sampling sites (private alleles) and to quantify the prevalence of such alleles within each area. Genetic population structure was modeled using STRUCTURE v2.3.4 (Pritchard et al., 2000). The model was run using locprior with one, two, and three possible populations (*K* = 1, *K* = 2, and *K* = 3). Selecting a model with a K value of one would indicate our sampling locations were from one panmictic population, two would mean that each site hosted a single distinct population, and three would imply that one or both aggregations were composed of multiple distinct populations. For each value of K, the model was run 10 times with a burn‐in of 10,000 iterations, followed by 1,000,000 iterations, and 10 iterations of each *K* scenario. STRUCTURE results were uploaded to Structure Harvester (Earl, 2012) where both Evanno's delta *K* and averaged maximum likelihood scores were calculated and used to determine the most likely value of K (Appendix S1: SI 2). After selecting the optimal *K* value, results were uploaded to CLUMPAK (Kopelman et al., 2015) to generate the consolidated plot.

In order to assess the likelihood of recent population expansions in Saudi Arabia or Tanzania, microsatellite loci were analyzed using the Microsoft Excel macro KGTESTS (Bilgin, 2007). A within‐locus *k*‐test was used to compare observed microsatellite allelic distributions with those expected under mutation–drift equilibrium. A negative value in the *k*‐test is indicative of population expansion while positive values suggest population stagnation (Reich et al., 1999). The significance of *k* was determined using a one‐tailed binomial test. In addition, an interlocus *g*‐test was used to compare observed versus expected allele size variances across all loci (Bilgin, 2007). This ratio is expected to be small in a recently expanded population in which allele genealogies show recent coalescence, but large in a population of constant size because of longer histories of variable mutation rates among loci. To determine the significance of the test, *g* values were compared to the expected 5% cut off of *g* under constant population size (Reich et al., 1999).

### 2.6 Mitochondrial marker analysis

Microsatellite makers are useful, but differences in the chosen loci and scoring biases make results difficult to compare among studies. Using the mitochondrial control region allows for the incorporation of sequences from published sources to build a global comparison. Mitochondrial sequences were aligned, edited, and trimmed using Geneious 8.1.6 (Biomatters Ltd.) and the original sequences were uploaded to GenBank (accession numbers: OL782199–OL782316). Whale shark control region sequences from previous studies (Djibouti, Qatar, Mozambique, Seychelles, Maldives, Western Australia, Philippines, Taiwan, Japan, Mexican Pacific, Mexican Atlantic; Castro et al., 2007; Meekan et al., 2017; Ramírez‐Macías et al., 2007; Schmidt et al., 2010; Sigsgaard et al., 2017; Vignaud et al., 2014; Yagishita et al., 2020) were incorporated into the alignment for subsequent analyses (Appendix S1: SI 3).

Most prior studies (Castro et al., 2007; Vignaud et al., 2014; Yagishita et al., 2020) have retained gaps in the alignment on the assumption that these regions represented bioinformative deletion/insertion mutations. These papers followed a dual approach using a fully gap inclusive “raw alignment” to build their haplotype networks and a gap‐reduced “modified alignment” for demographic analyses. The modified alignment reduced contiguous, nonvariable gap regions to a single transition, effectively assuming they were caused by a single indel. In contrast, at least one paper has elected to simplify the alignment methodology by excluding gaps entirely (Meekan et al., 2017). To facilitate comparison to all previous works and investigate the bioinformatic value of these regions, the Roehl data files for the haplotype network constructions were generated with the full gaps (raw alignment), reduced gaps (modified alignment), and without gaps. Haplotype networks were constructed for both the fully gap‐inclusive and ‐exclusive data, while demographic analyses were calculated for all three alignments. Haplotype statistics and Roehl data were generated using DnaSP version 5.0 (Librado & Rozas, 2009) for subsequent haplotype network analysis. Within the Roehl data file, all the nonvariable sites in the alignment were discarded. Evolutionary relationships among whale shark mitochondrial haplotypes were assessed with a median‐joining network, constructed with the program NETWORK version 4.5.1.0 (www.fluxus‐engineering.com/network_terms.htm) using default settings.

In Arlequin v3.5 (Excoffier & Lischer, 2010), each sampling site was treated as a population and genetic structure was tested with analysis of molecular variance (Nei & Jin, 1989) with 10,000 permutations, using the T92+G substitution model (Tamura, 1992) with a gamma shape parameter of 0.48 (Appendix S1: SI 4). This model aims to estimate the genetic divergences between pairwise samples using *F*_ST_ based on haplotype frequencies and molecular divergence. The resulting *p*‐values were compared to both a standard α (.05) and a multiple‐comparisons‐corrected α (.01) (Narum, 2006). Several metrics of genetic diversity, including the haplotype diversity (*h*) and nucleotide diversity (*π*), were calculated for each location, and all samples combined, using Arlequin v 3.5 (Excoffier & Lischer, 2010). Haplotype diversity within the Saudi Arabian Red Sea was also calculated and compared among years using a Mann–Kendall trend test (Kendall, 1955; Mann, 1945) to assess recent temporal fluctuation in the genetic makeup of the aggregation. Longer term population trends were visualized using Bayesian skyline plots (BSPs) generated for the overall dataset, for the Indo‐Pacific, and for the Atlantic. Several neutrality statistics including Tajima's *D*, Fu's *F*, and Harpending's raggedness index (HRI) were calculated (again using Arlequin v 3.5; Excoffier & Lischer, 2010) to detect evidence of historical population expansions within each location, and for all samples combined. Fu's *F* is known to be extremely sensitive and possibly confounded by species‐specific recombination rates (Rozas & Calafell, 2008). These rates are unknown for the whale shark, making Fu's *F* somewhat unreliable for this species. It has been included to facilitate comparison with previous work.

## 3 RESULTS

### 3.1 Data filtering

Of the 22 candidate microsatellite markers initially considered for this study, six loci (Rhin_t_04, Rtyp1, Rtyp3, Rtyp4, Rtyp8, and Rty_15) could not be successfully amplified. Another three loci (Rty_16, Rhin_t_28, and Rhin_t_46) contained null alleles, one locus (Rtyp7) showed strong evidence of linkage with multiple other loci, and one locus (Rhin_t_13) violated the assumptions of the Hardy–Weinberg equilibrium. The remaining 11 markers consisted of 2 from Ramirez‐Macias et al. (2009) and 9 that were designed for the present study (Table 1). The probability of identity for these markers ranged from 0.03 to 0.59 with a combined probability of 7.3 × 10^−9^ when all 11 loci were used together.

**TABLE 1:** Subset of 11 microsatellite markers used for the analysis of 156 individual whale sharks (Saudi Arabia: 84, Tanzania: 72), including the number of alleles per locus (*N*_a_), the observed heterozygosity (*H*_o_), the expected heterozygosity (*H*_e_)

Locus	Size range (bp)	*N*_a_	*H*_o_	*H*_e_	*k* Test
Rty_18*	175–185	4	0.475	0.451	−349.13
Rty_38*	172–185	7	0.740	0.728	63.82
Rhin_t_03	247–255	5	0.639	0.645	1183.79
Rhin_t_05	220–248	12	0.835	0.863	−562.74
Rhin_t_07	264–286	10	0.821	0.831	195828.79
Rhin_t_10	144–154	5	0.661	0.667	−30.96
Rhin_t_11	143–149	3	0.242	0.239	−12.12
Rhin_t_30	321–336	6	0.075	0.073	214.22
Rhin_t_31	152–164	4	0.412	0.427	−28.70
Rhin_t_32	115–123	5	0.631	0.702	3230.23
Rhin_t_47	117–139	7	0.590	0.620	−1768.07
11 Total loci					*p*‐Value = .46

Note

Intralocus variance (*k*) values are provided for each marker, an interlocus variance (*g*) is provided for the study as a whole. Locus names with * are sourced from Ramirez‐Macias et al. (2009).

A total of 239 tissue samples (102 from Saudi Arabia, 137 from Tanzania) were genotyped, revealing 87 duplicates (18 from Saudi Arabia, 65 from Tanzania). Comparing the two highest quality samples from a subset of duplicated sharks (*n* = 35) produced a genotyping error rate of 1.1% per locus. The 156 highest quality, unique genotypes were retained for further analysis. This filtered dataset included 84 unique individuals from Saudi Arabia (40 females, 33 males, 11 unknown) and 72 from Tanzania (12 females, 58 males, 2 unknown). Sharks sampled in Saudi Arabia had an average estimated total length of 4.04 m (range: 3–7 m) while those in Tanzania averaged 5.99 m (range 3.5–8 m). As of 2015, there were 136 photo‐identified sharks from Saudi Arabia (Cochran et al., 2016), including 81 of the sharks sampled in this study. Using the same 2015 end‐date for Tanzania (Prebble et al., 2018) yields 139 photo‐identified sharks, including 65 individuals from the present study.

### 3.2 Comparing Saudi Arabia and Tanzania

The microsatellite datasets from Saudi Arabia and Tanzania had an allelic richness of 5.30 and 5.07, respectively. No distinct private alleles were found at either aggregation. Given the number of sampled individuals, private alleles at either site likely have low prevalence (mean expected frequency of private alleles is 0.0087), if they exist at all. Genetic differentiation between the two sampling sites was small (microsatellite *F*_ST_ = 0.0028) indicating connectivity sufficient to maintain population homogeneity. This was also supported by Shannon's Information Index, which showed that more than 98% of the total information derived from individual variation within the two aggregation sites, as opposed to less than 2% from difference between them. Observed heterozygosity estimates at each site (Saudi Arabia: 0.55, Tanzania: 0.56) were similar (χ^2^ test, *p* > .05) to each other and to expected values (0.57 in both cases). Results from the program STRUCTURE suggested *K* = 1 (one source population for both sites) as the most likely configuration (Appendix S1: SI 2).

### 3.3 Trends in genetic diversity

The KGTESTS analyses of the microsatellite data also indicated population stability for both the Saudi Arabian and Tanzanian aggregations. Within‐locus *k*‐tests produced a mixture of positive and negative values for different markers (Table 1) and did not reveal any significant evidence of historical population changes at either aggregation (*p *= .46). Similarly, the interlocus *g*‐test produced a value of 3.46, far above the modeled 5% cutoff of 0.19. In the Red Sea, population stability was further supported by quantitating genetic diversity within the Saudi Arabian aggregation site over the 6‐year sampling period. Allelic richness showed little year‐to‐year variation (range: 2.93–3.16) and no consistent directional trend (Mann–Kendall trend test, tau = 0.316, *p* > .05) (Table 2). This pattern of stability was also maintained when only the first (2010, allelic richness: 4.30) and final (2015, allelic richness: 4.32) sampling years were compared, confirming that genetic diversity remained constant over the 6‐year period and that low sample sizes in specific years did not bias the overall pattern. Mitochondrial analysis yielded similar results with haplotype diversity remaining stable in Saudi Arabia from 2010 (haplotype diversity: 0.859) to 2015 (0.940) (Mann–Kendall trend test, tau = 0.600, *p* > .05) (Table 2).

**TABLE 2:** Temporal genetic diversity of Saudi Arabian Red Sea whale sharks

	2010	2011	2012	2013	2015
**Microsatellite *N***	30	26	9	7	19
Allelic Richness	3.04	3.01	2.93	3.04	3.16
*Allelic Richness	4.3				4.32
**Mitochondrial *N***	19	17	8	8	16
Haplotype Diversity	0.859	0.844	0.900	0.867	0.940

Note

Microsatellite *N* is the number of individuals for which microsatellite data were available from each season. Sample size fluctuated among years, which can affect the calculations for allelic richness. To account for this, change in allelic richness was also assessed using only data from the first and final seasons (noted with an *). Mitochondrial *N* is number of individuals for which mitochondrial sequence data were available. All analyses indicated population stability at this site over the study period.

### 3.4 Global structure

An initial alignment of 816 mitochondrial sequences was created from 699 publicly available sequences and 117 sequences from this study (Tanzania *n* = 57, Saudi Arabia *n* = 60). Previous work included 26 sequences from the Saudi Arabian Red Sea (Vignaud et al., 2014; Yagishita et al., 2020); we have updated the sequencing for 16 of these individuals and sourced another 8 directly from online databases. In the main text we have largely focused on the gap‐inclusive results while the gap‐exclusive data are fully available in the appendix. A final whale shark control region fragment of 673 bp was used for subsequent analyses, encompassing 816 individuals from 13 locations (Appendix S1: SI 3). The alignment contained 328 variable sites, including sequence gaps, resulting in 192 identified haplotypes, an average haplotype diversity of 0.944 (range: 0.85–1.00), and an average nucleotide diversity of 0.12 (range: 0.05–0.17).

**FIGURE 2:**
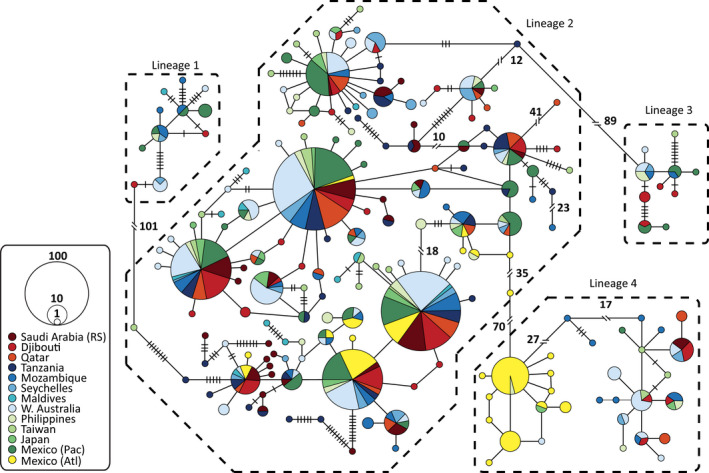
Relationships of *Rhincodon typus* haplotypes, from 13 different geographic locations (color in legend), in median‐joining network created using mitochondrial whale shark control region sequences where gaps were considered informative. Each circle represents a unique haplotype and is proportional to total haplotype frequency. Branches connecting circles represents a single nucleotide substitution; black cross‐bar represents an additional nucleotide substitution; black double slash bars represent more than 10 nucleotide substitutions (exact numbers noted). Areas encompassed by dashed lines represent four putative lineages. Atl, Atlantic; Pac, Pacific; RS, Red Sea

**FIGURE 3:**
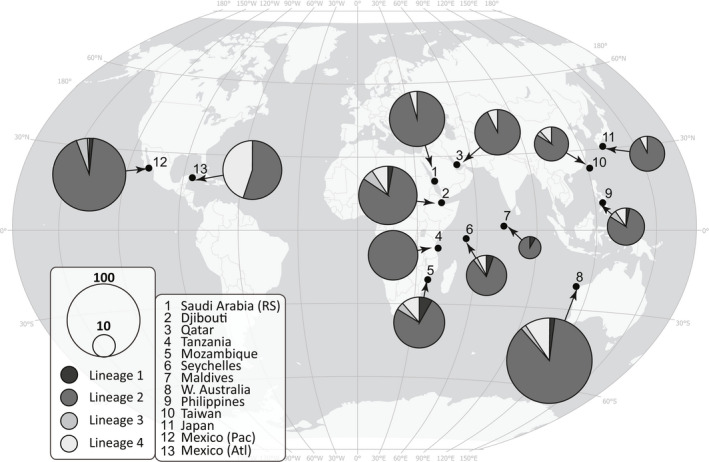
Locations from this study and mitochondrial sequences sourced from previous publications, and haplotype lineage composition of *Rhincodon typus* individuals at each aggregation site (numbers in legend). Circles display the lineage composition of individuals sequenced from that aggregation (colors representing four putative haplotype lineages in legend) and circle size is proportional to the number samples. Atl, Atlantic; Pac, Pacific; RS, Red Sea

The haplotype network was divided into four major lineages with no clear pattern in how haplotypes were distributed among aggregation sites (Figure 2). The one exception to this general lack of geographic structure was the disproportionate grouping of Atlantic samples within lineage 4 (Figure 3). This was also reflected in the statistical analysis of the mitochondrial sequences, which revealed relatively strong (mitochondrial *F*_ST_ = 0.0532–0.1456) and significant (*p* < .05) differences between the Atlantic samples and those from the Indo‐Pacific sites (Table 3). The analysis also showed some smaller (mitochondrial *F*_ST_ = −0.0042–0.023), but still statistically significant (*p* <.05), differences among Indo‐Pacific aggregations. The Maldives exhibited some stronger differences to other Indo‐Pacific sites (mitochondrial *F*_ST_ = 0.0325–0.0482), but this could be attributed to the limited number of samples available from that region (Table 3). Removing sequence gaps did not substantially affect the results (Appendix S1: SI 5). Overall, the gap‐inclusive and ‐exclusive networks exhibited similar geographic distributions of haplotypes and patterns of population structure (Table 3, Appendix S1: SI 6).

**TABLE 3:** Population pairwise *F*_ST_ and *p*‐values based on haplotype frequencies (gaps considered informative)

	Saudi Arabia (RS)	Djibouti	Qatar	Tanzania	Mozambique	Seychelles	Maldives	W. Australia	Philippines	Taiwan	Japan	Mexico (Pac)	Mexico (Atl)
Saudi Arabia (RS)		–	–	–	–	–	–	–	–	–	–	++	++
Djibouti	0.0001		++	–	–	+	–	+	–	+	–	++	++
Qatar	0.0053	0.0183		–	–	–	+	–	–	–	–	–	++
Tanzania	−0.0012	0.0071	0.0013		–	–	–	–	–	–	–	–	++
Mozambique	0.0004	0.0068	0.0052	−0.0011		–	–	–	–	–	–	–	++
Seychelles	0.008	0.0133	0.0093	0.0046	0.0045		–	–	–	–	–	–	++
Maldives	0.0086	0.0051	0.0325	0.0057	0.0058	0.0142		+	–	–	–	–	++
W. Australia	0.0022	0.0092	0.0014	0.0055	0.0028	0.006	0.023		–	–	–	–	++
Philippines	0.0015	0.0016	0.0077	−0.0024	−0.0081	0.0004	−0.0031	0.0032		–	–	–	++
Taiwan	0.0088	0.019	0.003	−0.0012	0.0027	0.0042	0.005	0.0077	0.0035		+	–	++
Japan	0.0025	−0.0008	0.0121	0.0082	−0.0054	0.0118	0.0071	0.0014	−0.0048	0.0164		–	++
Mexico (Pac)	0.0106	0.0139	0.0007	0.006	0.0052	0.0106	0.0246	0.0041	0.0048	0.0062	0.0059		++
Mexico (Atl)	0.0791	0.068	0.0974	0.0747	0.0684	0.0742	0.0702	0.0791	0.0532	0.0873	0.0658	0.0797	

Note

Not significant; + *p* < .05; ++ *p* < .01 (corrected for multiple comparison).

Abbreviations: Atl, Atlantic; Pac, Pacific;RS, Red Sea.

### 3.5 Demographic history

The gap‐inclusive network was characterized by a highly reticulated structure, which is an indication of population stability (Figure 2). This was also reflected in the neutrality statistics. Tajima's *D* was nonsignificant for most study sites (Table 4) and for the Indo‐Pacific overall (Tajima's *D* = −1.23, *p* > .05). Fu's *Fs* was nonsignificant for every individual study site but was significant for the Indo‐Pacific as a whole (Fu's *Fs* = −23.31, *p* <.05). Both tests were nonsignificant for the Mexican Atlantic, as were the HRI values for all sites and both ocean basins. Unlike population structure, the demographic history results changed when the gap data were reduced or removed. The different lineages within the haplotype network tended to form star‐shaped clusters more indicative of recent population expansions (Ferreri et al., 2011). Tajima's *D* was negative for all Indo‐Pacific study sites, and significantly so for most (Appendix S1: SI 7 and 8). More importantly, Tajima's *D* was both negative (−1.45) and significant (*p* < .05) when these sites were pooled together and tested as a whole. Fu's *Fs* results were more regionally varied, but only Tanzania showed significant evidence of a regional expansion (*Fs* = −10.32, *p* < .01) and only when gaps were removed entirely. Despite these varied regional results, Fu's *Fs* indicated significant historical expansion of the Indo‐Pacific population overall for both the gap‐reduced (*Fs* = −23.10, *p* < .05) and gap‐exclusive (*Fs* = −23.41, *p* < .05) alignments. The Mexican Atlantic never showed significant deviation from population neutrality. The HRI was not consistently significant for any sampling site, and was never significant for the Indo‐Pacific collectively.

**TABLE 4:** Measures of genetic diversity at each location (gaps considered); number of sequences (*n*), number of haplotypes (*N*_hp_), haplotype diversity (*h*), and nucleotide diversity (*π*), Tajima's *D*, Fu's *Fs*, and Harpending's raggedness index (HRI)

Sampling locations	*n*	Genetic diversity			Neutrality tests		Mismatch distribution (HRI)
		*N*_hp_	*h*	*π*	Tajima's *D*	Fu's *Fs*	
Saudi Arabia (RS)	68	31	0.93 ± 0.02	0.07 ± 0.04	−1.61^+^	0.16^NS^	0.02^NS^
Djibouti	77	31	0.93 ± 0.01	0.13 ± 0.06	−1.23^NS^	8.31^NS^	0.01^NS^
Qatar	54	20	0.90 ± 0.03	0.08 ± 0.04	−1.19^NS^	5.34^NS^	0.02^NS^
Tanzania	57	33	0.96 ± 0.01	0.05 ± 0.03	−0.62^NS^	−4.87^NS^	0.01^NS^
Mozambique	62	33	0.96 ± 0.01	0.17 ± 0.08	−0.51^NS^	5.18^NS^	0.01^NS^
Seychelles	38	21	0.95 ± 0.02	0.13 ± 0.06	−1.60^NS^	5.14^NS^	0.02^NS^
Maldives	12	12	1.00 ± 0.03	0.13 ± 0.06	−1.91^++^	−1.42^NS^	0.02^NS^
W. Australia	162	48	0.92 ± 0.01	0.10 ± 0.05	−1.34^NS^	4.08^NS^	0.01^NS^
Philippines	31	22	0.97 ± 0.02	0.15 ± 0.07	−1.60^+^	2.81^NS^	0.01^NS^
Taiwan	26	21	0.97 ± 0.03	0.12 ± 0.06	−1.13^NS^	−0.26^NS^	0.01^NS^
Japan	28	15	0.95 ± 0.02	0.09 ± 0.04	−1.44^NS^	−3.45^NS^	0.02^NS^
Mexico (Pac)	121	33	0.93 ± 0.01	0.11 ± 0.05	−1.50^+^	11.22^NS^	0.01^NS^
Mexico (Atl)	80	24	0.85 ± 0.03	0.17 ± 0.08	3.72^NS^	20.16^NS^	0.08^NS^
Indo‐Pacific	736	178	0.94 ± 0.00	0.10 ± 0.05	−1.23^NS^	−23.31^+^	0.01^NS^
Overall	816	192	0.94 ± 0.00	0.12 ± 0.06	−0.89^NS^	−23.31^+^	0.01^NS^

Note

^+^0.01 < *p* < .05; ^++^ *p* < .01 (corrected for multiple comparisons).

Abbreviations: Atl, Atlantic; NS, not significant; Pac, Pacific; RS, Red Sea.

All of the BSPs, regardless of gap treatment or basin grouping, showed long‐term stability of the targeted population followed by a recent expansion (Appendix S1: SI 9). However, the broad trends proceeding the population expansion varied. For instance, the fully gap‐inclusive plots for both the overall data and the Indo‐Pacific showed a gradual, long‐term population decline while the gap‐reduced and gap‐exclusive alignments showed a much more abrupt bottlenecking event (Appendix S1: SI 9). The Atlantic showed a bottleneck and subsequent recovery in all of the alignments, but in all cases the difference between the minimum and maximum population size was less pronounced than in the Indo‐Pacific, indicating relative stability in the Atlantic population overall.

## 4 DISCUSSION

This study provides the first mitochondrial and microsatellite sequences from the Mafia Island aggregation site in Tanzania and nearly quadruples the sample size from the Shib Habil aggregation site in the Saudi Arabian Red Sea. Both Tanzania and Saudi Arabia were shown to attract whale sharks from a single, basin‐scale population that extends throughout the Indo‐Pacific. Nucleotide and haplotype diversity were both stable at the Saudi Arabian aggregation site over the 6‐year sampling period, a direct contrast to the declines reported in Western Australia (Vignaud et al., 2014). Globally, our results corroborate the division of whale sharks into distinct Atlantic and Indo‐Pacific populations, but contrast recent population expansions inferred by previous work (Vignaud et al., 2014; Yagishita et al., 2020).

### 4.1 Comparing Saudi Arabia and Tanzania

Allelic richness was similar between Saudi Arabia and Tanzania, although the richness metrics for Saudi Arabia were slightly higher than those from previous work (Vignaud et al., 2014). This is likely due to differences in sample sizes, chosen microsatellite loci, or a combination of both among different studies. All analyses (*F*_ST_, private allele assessment, both haplotype networks, and STRUCTURE analysis) indicate genetic exchange between the sharks from the Tanzanian site and from the Saudi Arabian site. This would imply that the sharks from both aggregation sites belong to the same population. Previous work has shown that sharks sampled at the Saudi Arabian site and aggregation sites along the east coast of Africa (Djibouti and Mozambique) are all grouped within the Indo‐Pacific population (Yagishita et al., 2020; Meekan et al., 2017; Vignaud et al., 2014). The results here demonstrate that the Tanzanian aggregation site, despite local residency of the sharks (Cagua et al., 2015; Rohner et al., 2020), is part of the same genetic population as neighboring aggregation sites along the east African coast.

The observation that whale sharks sampled in Saudi Arabia and Tanzania are members of the same population is particularly interesting as the movement ecologies and demography at the two aggregation sites are quite distinct. Sharks at the two sites exhibit markedly different residency behaviors (Cagua et al., 2015; Cochran et al.,^2019^), movement patterns (Berumen et al., 2014; Rohner et al., 2020), and size/sex demographics (Cochran et al., 2016; Rohner et al., 2015). Saudi Arabia hosts a seasonal aggregation (Cagua et al., 2015) that attracts relatively small (average total length: 4.04 m), juvenile sharks of both sexes in roughly equal numbers (Cochran et al., 2016). Satellite telemetry from this site has shown that the sharks disperse hundreds of kilometers across the wider Red Sea and into the Indian Ocean after the aggregation season ends (Berumen et al., 2014). Conversely, Tanzania hosts a year‐round resident aggregation (Cagua et al., 2015) of comparatively large (average total length: 5.99 m) juvenile males (Rohner et al., 2015) that seasonally move only a few tens of kilometers between near‐shore and off‐shore habitats (Rohner et al., 2020). The results here suggest that these differences are not driven by population‐level genetic variation between the two sites but may instead be examples of behavioral plasticity in response to each aggregation's local environment. Protection legislation and other conservation actions should account for these differences because, despite genetic homogeneity within the Indo‐Pacific, there is unlikely to be a “one‐size‐fits‐all” approach to managing whale shark aggregations.

### 4.2 Trends in genetic diversity

Based on the KGTEST results, both the Saudi Arabian and Tanzanian populations appear to be stable over relatively short time scales. Similarly, both allelic richness and haplotype diversity at the Saudi Arabian aggregation site appear to have been stable from 2010 through 2015. These results are counter to the declines in diversity reported in Western Australia from 2007 to 2012 (Vignaud et al., 2014). Based on present results and previous studies (Meekan et al., 2017; Vignaud et al., 2014; Yagishita et al., 2020), sharks sampled in Western Australia and Saudi Arabia are both part of a single Indo‐Pacific population. The declines in Western Australia, and the absence of declines in Saudi Arabia, suggest that the former's losses of genetic diversity are the result of local processes as opposed to global‐ or population‐scale phenomena (Vignaud et al., 2014). It is also possible, given the distance between the two sites, that there is a time lag between impacts on one portion of the population affecting the other. If this is the case, then continued monitoring in Saudi Arabia might eventually be expected to show declines similar to those reported from Western Australia.

Several potential threats may disproportionately affect whale sharks from Western Australia when compared to those from the Saudi Arabian Red Sea, including natural predation as well as anthropogenic pressures. Two predators are known to attack and occasionally kill whale sharks: the killer whale *Orcinus orca* (O'Sullivan, 2000) and the white shark *Carcharodon carcharias* (Fitzpatrick et al., 2006). Both are relatively common along the western coast of Australia (McAuley et al., 2017); Pitman et al., 2015), but rare (Notarbartolo di Sciara et al., 2017) or absent (Golani & Bogorodsky, 2010) within the Red Sea. This difference is reflected in the prevalence and types of scars reported from whale sharks in the Saudi Arabian Red Sea and Western Australia. Scarring consistent with boat strikes are commonly seen at both sites (Cochran et al., 2016; Speed et al., 2008), although scars directly attributed to boats are less frequent in Western Australia than in Saudi Arabia (Lester et al., 2020). Conversely, bite marks have not been documented in Saudi Arabia (Cochran et al., 2016) while in Western Australia 4.8%–11% of individuals had predator bite scars (Lester et al., 2020; Speed et al., 2008). In addition to natural predators, Western Australia is also in relatively close proximity to several historical whale shark fisheries (Alava et al., 1997; Chen et al., 1997; White & Cavanagh, 2007), and at least one that still actively targets these animals (Li et al., 2012). In contrast, there are no known current or former fisheries dedicated to landing whale sharks within the Red Sea.

Conversely, other threats (particularly boat strike) are arguably more prevalent in the Red Sea given its status as one of the world's busiest shipping lanes (Stevens, 2007). The use of spotter planes to direct tourism boats to whale shark locations within Ningaloo Marine Park may also decrease the threat of collisions in that immediate area. At Shib Habil, Saudi Arabia, tourism operations rely exclusively on boat‐based surveys, increasing the potential risk of boat strike (Lester et al., 2020). Furthermore, the loss of genetic diversity reported from Western Australia does not necessarily correspond to declines in local whale shark abundance (Vignaud et al., 2014). While the different trends in genetic diversity in Saudi Arabia and Western Australia certainly warrant additional investigation, conclusions from both sites are based on only 6 years of data. These are relatively short time series when compared to the potential life span of the species (Ong et al., 2020). Continued sampling at both sites and at other aggregations could substantially improve this comparison and help to identify the exact causes for the declines in genetic diversity shown in Western Australia (Vignaud et al., 2014).

### 4.3 Global structure

The haplotype networks all revealed relatively small, but statistically significant differences between Indo‐Pacific aggregation sites (Table 3, Appendix S1: SI 6). This is consistent with previous research on whale shark population genetics (Vignaud et al., 2014; Yagishita et al., 2020). Given the low *F*_ST_ values, previous studies have simply dismissed these differences as either ecologically negligible or (in the case of the Maldives) artifacts of small sample sizes (Vignaud et al., 2014; Yagishita et al., 2020). This conclusion is also supported by more general guidelines for interpreting *F*_ST_ that describe values below 0.05 (as is the case for all Indo‐Pacific comparisons in the present study) as relatively weak indicators of population structure (Hartl et al., 1997). While these differences might warrant further investigation, they are not sufficient evidence to justify further subdivision of the Indo‐Pacific population.

Most importantly, the much larger differences recorded between the Atlantic and Indo‐Pacific support the existence of two basin‐scale whale shark populations. The first global haplotype network for whale sharks was built using 69 samples from 6 aggregations and split the species into distinct Atlantic and Indo‐Pacific populations (Castro et al., 2007). This original network was updated in 2014 (Vignaud et al., 2014), 2017 (Meekan et al., 2017), (Yagishita et al., 2020), and again in the present study using 816 samples from 13 sites. Despite more than an order of magnitude increase in sample size and the use of three different alignment methodologies, the division of whale sharks into Atlantic and Indo‐Pacific populations has been consistent across studies and is further confirmed by our results. Given the robustness of the two‐population model for global whale shark population dynamics, it seems unlikely that additional sampling from novel sites will significantly alter these results. Revealing finer scale differentiation among distant sites within each basin may require a fundamental shift in methodology, either toward single‐nucleotide polymorphisms or population genomics.

The relative lack of genetic structure among global whale shark aggregations is unusual when compared to many other pelagic mega‐planktivores. There is evidence for genetic differentiation among of several cetaceans species (Jackson et al., 2014; Sremba et al., 2012) and both manta species (*Mobula birostris* and *Mobula alfredi*) (Stewart et al., 2016; Venables et al., 2021) within ocean basins. However, the lack of basin‐scale differentiation appears to be a shared trait among the filter‐feeding sharks. The basking shark *Cetorhinus maximus* (Rus Hoelzel et al., 2006) and the megamouth shark *Megachasma pelagios* (Liu et al., 2018) are both characterized by a single, globally distributed population making both of these species less divergent than the whale shark. While recent research has suggested some level of genetic structure for the basking shark in the northeast Atlantic, a single panmictic population remains the prevailing view (Lieber et al., 2020). If correct, the single population model for both the basking and megamouth sharks suggests a clear difference in the recent dispersal patterns of the three planktivorous shark species. This difference may derive from the whale shark's clear preference for the tropical and warm temperate seas (Rowat & Brooks, 2012), where several landmasses act as barriers to migration and gene flow. The basking and megamouth sharks are more tolerant of colder waters and are known to exhibit extended periods of residency in the deep ocean which may alleviate some barriers to movement and genetic exchange across basins. Alternatively, the global homogeneity found in these two species could be due to historical, transarctic connectivity during the late Pleistocene (Lieber et al., 2020).

### 4.4 Demographic history

Two previous studies used neutrality statistics and other methods to detect fluctuations in the Indo‐Pacific whale shark population (Vignaud et al., 2014; Yagishita et al., 2020). Both of these studies reduced gap regions within their mitochondrial alignments for these analyses, effectively assuming that contiguous gaps resulted from singular events rather than independent mutations (Simmons & Ochoterena, 2000), and both found strong evidence for population expansion (Vignaud et al., 2014; Yagishita et al., 2020). These previous results are consistent with both the gap‐exclusive and gap‐reduced mitochondrial networks from the present study (Appendix S1: SI 5). The Indo‐Pacific Tajima's *D* and Fu's *Fs* were significantly negative indicating recent population expansion in both cases (Appendix S1: SI 7 and 8) The evidence for population expansion became much weaker when contiguous gaps were analyzed. The Indo‐Pacific Tajima's *D* was not significant. Fu's *Fs* remained significantly negative, but this may be attributed to the metric's oversensitivity or to the potential confounding effects of species‐specific recombination rates (Rozas & Calafell, 2008). Similarly, HRI values were not significant for all sites. This is consistent with Yagishita et al. (2020), who found nonsignificant HRI values for whale sharks in the Atlantic, where most other metrics indicated a stable population. The alignment differences in the BSPs were less pronounced, with all groupings showing some evidence of population expansion. However, the abruptness and magnitude of the population declines preceding the recent expansions differed between gap treatments and between ocean basins. These mixed results raise some doubt on the recent population expansions proposed by earlier studies (Vignaud et al., 2014; Yagishita et al., 2020).

The differences between the gap‐inclusive, ‐reduced, and ‐exclusive networks also highlight the potential effects that relatively minor methodological changes can have on final results. The gaps themselves were highly varied and their positions within the alignment were consistent across samples from different studies and geographic regions. This suggests that they are unlikely to be artifacts of sequencing or analytical methodologies and are most likely bioinformative collections of deletion mutations. Regardless, disentangling whale shark demographic history will require additional sampling and more advanced sequencing methods, but an improved understanding of past fluctuations in whale shark abundance may help conservation efforts to gauge and manage current or future population changes in the face of anthropogenic pressures.

## 5 CONCLUSIONS

This paper adds samples from whale sharks in two understudied regions, describes new microsatellite markers, and provides a point of comparison to previous results from other aggregations (Vignaud et al., 2014). In addition, most of the new mitochondrial sequences from this study have been directly linked to identification photos for sampled sharks (Appendix S1: SI 10 and 11), with accession numbers added to the online profile of each individual (sharkbook.ai). Linking the genetic and photographic datasets in this way can simplify future analysis and facilitate multi‐method studies. The results here strongly suggest that the Saudi Arabian and Tanzanian aggregation sites are part of a larger Indo‐Pacific population despite the two sites being over 4500 km apart, showing no direct evidence of connectivity (through photo‐ID or tagging studies), and exhibiting markedly different local behavioral ecologies. This would seem to indicate that basin‐scale genetic exchange occurs later in the whale shark's life history or that aggregations are “seeded” with the offspring of a more interconnected, adult population. In terms of conservation, this would mean that local protections at aggregation sites are important but probably insufficient as the sole means of managing a largely pelagic species. Effective high seas protections will likely be crucial for the long‐term survival of whale shark and other species that frequently move through international waters (Queiroz et al., 2019). However, the development of such protections is currently hindered by the inability to reliably find mature sharks and the relatively short‐term deployment capabilities of most current satellite transmitters. Until direct, long‐term monitoring of large whale sharks becomes readily available, genetic inference remains the best method for mapping the species’ global patterns of dispersal.

The broad division of whale sharks into two basin‐scale populations (i.e., Indo‐Pacific and Atlantic) appears to be robust and resolving finer scale differences among aggregating subpopulations (e.g., within the Indo‐Pacific) may require researchers to move beyond microsatellite and mitochondrial markers and on to more comprehensive sequencing methods. The recent publication of complete mitochondrial‐genome (Alam et al., 2014) and whole‐genome (Hara et al., 2018; Read et al., 2017; Weber et al., 2020) assemblies for the whale shark represents an important first step away from population genetics and toward population genomics. Improved scientific understanding of the genetic structure and long‐term patterns of connectivity among whale shark aggregation sites will be vital to the effective global conservation of this endangered species.

## CONFLICT OF INTEREST

The authors declare no competing interests.

## AUTHOR CONTRIBUTIONS

**Royale S. Hardenstine:** Conceptualization (equal); Data curation (equal); Formal analysis (supporting); Methodology (equal); Resources (supporting); Visualization (equal); Writing – original draft (equal). **Song He:** Conceptualization (supporting); Data curation (equal); Formal analysis (lead); Methodology (equal); Visualization (equal); Writing – original draft (supporting). **Jesse E. M. Cochran:** Conceptualization (supporting); Methodology (equal); Resources (equal); Writing – original draft (equal); Writing – review & editing (lead). **Camrin D. Braun:** Resources (equal); Writing – review & editing (equal). **E. Fernando Cagua:** Resources (equal); Writing – review & editing (equal). **Simon J. Pierce:** Funding acquisition (equal); Project administration (equal); Resources (equal); Writing – review & editing (equal). **Clare E. M. Prebble:** Data curation (supporting); Resources (equal); Writing – review & editing (equal). **Christoph A. Rohner:** Data curation (supporting); Resources (equal); Writing – review & editing (equal). **Pablo Saenz‐Angudelo:** Methodology (equal); Resources (equal); Writing – review & editing (equal). **Tane H. Sinclair‐Taylor:** Resources (equal); Writing – review & editing (equal). **Gregory B. Skomal:** Funding acquisition (equal); Writing – review & editing (equal). **Simon R. Thorrold:** Funding acquisition (equal); Writing – review & editing (equal). **Alexandra M. Watts:** Formal analysis (supporting); Writing – review & editing (equal). **Casey J. Zakroff:** Methodology (supporting); Writing – review & editing (equal). **Michael L. Berumen:** Conceptualization (equal); Funding acquisition (equal); Project administration (equal); Supervision (lead); Writing – review & editing (equal).

## Supporting information

Supplementary Material

## Data Availability

All microsatellite genotypes and primers have been deposited onto Dryad (https://doi.org/10.5061/dryad.sqv9s4n58). All mitochondrial sequences are available on GenBank (accession numbers: OL782199–OL782316). If available, the accession number for an individual shark will be included on their respective Wildbook for Whale Sharks profile.
