# Corrigendum

**DOI:** 10.1002/ece3.8727

**Published:** 2022-03-23

In the recent article by Hardenstine et al. (2022), a spelling error was introduced to the surname of the ninth author. The correct author surname is Pablo Saenz‐Agudelo.

This has now been corrected in the article.

The authors apologize for this error.
