# Supplementary material for: Pieces in a global puzzle: Population genetics at two whale shark aggregations in the western Indian Ocean
Source: Ecol Evol. 2022 Jan 25;12(1):e8492. doi: 10.1002/ece3.8492 (PMC8796955; doi:10.1002/ece3.8492)
Supplement: Supplementary file 1 — Supplementary Material [file ECE3-12-e8492-s001.pdf]

APPENDIX 1: SUPPLEMENTARY INFORMATION

Supplementary Information 1: Original set of 22 microsatellite loci tested for use in this study. *Loci from Schmidt et al. 2009, **loci from Ramirez-Macias et al. 2008, others were designed for this study.

Locus	Annealing Temp	Repeat	Primer Sequence
Rtyp1*	76.2	$(TG)_2TC(TG)_6TC(TG)$… $(TG)_9$…$(TG)_9TG(TG)^7$	F: AGGGGAGTGAATCTGTGGAAGTC<br>R: GTGTCTTCGCAGCAAACATCGTCTCAGTG
Rtyp3*	72.3	$(TG)_{14}$…$(TG)_5$…$(TG)_4$	F: GTTCAAATAGTGACTGGATGGAGAATGC<br>R: GTGTCTTGGATGCAACTAACATACACATGTAATATGG
Rtyp4*	74.8	$(CT)_3$…TTTTCTGT $(CT)_{14}GTCT$	F: TGGCGATGGTCTAACTTACATGAGC<br>R: GTGTCTTTCCGGACTTCATCACCCTAACATG
Rtyp7*	71.4	$(CA)_3TG(CA)_3TA(CA)_3$ $(CT)_4CC(CA)_{19}$	F: TGTACCTGTTGTATAGCATTGGAAGG<br>R: GTGTCTTGGGATTTATAAATAGCCACATTGACTG
Rtyp8*	73.4	$(CA)_3TGT(GC)_4(CA)_9TACA$	F: CGATTGGTTAACTAAGTCAGAGTATGG<br>R: GTGTCTTCGAAGTCTTTGCCCACTCACTTAAC
Rty_15**	63.8	$(CA)_{19}$…$(CA)_5CT(CA)_8$…$(CA)_{22}$	F: CTGTCGCCTGTCAGATGTTG<br>R: GGACTGGAAGCTGGTCTTTG
Rty_16**	61.0	$(G)_{11}$ $AAC(AG)_{20}$	F: ACGACCACTGGGCATTACAG<br>R: AAGGTTTAATATCCCTAGTTCAGACA
Rty_18**	64.0	$(AC)_{13}$	F: CAAAAACGCAGACCCATGTA<br>R: CACCAATGAACTCTGCTGGA
Rty_38**	64.2	$(CA)_{13}$	F: CGGGTCTACAGCAGGTGAAT<br>R: GACTCCAGACCCACAGCAAT
Rhin_t_03	61.0	$(AC)_{15}$	F: CTTGGCCTGCTGTGTTCATC<br>R: TATGTGCGTATAGGTGTGGC
Rhin_t_04	64.3	$(AC)_{16}$	F: TGAATCAGTGTGTAACGCGC<br>R: CGTGTATCCCTTGCGTGATAG
Rhin_t_05	64.7	$(AC)_{23}$	F: TGTGTTAGAACTCTGTGTCTGC<br>R: GCTCCATCCAGTGCACTAATTC
Rhin_t_07	65.4	$(AC)_{16}$	F: AGTGGAAGAACGAAACCTCTG<br>R: CCTCACAAATTTCACACGTGC
Rhin_t_10	59.6	$(AC)_{26}$	F: ACCCATCTCCCAACACCTTC<br>R: CTGACTGTGTGTGCGTACTC
Rhin_t_11	65.8	$(AC)_{22}$	F: TTATGAGCTGGGACCTGAGC<br>R: CACATCAAGGCCTCTTCGTG
Rhin_t_13	64.3	$(AC)_{18}$	F: TGCGTGTGTGAGAGAAGGG<br>R: TGTGTTGTCTCTCTGCCCAG
Rhin_t_28	67.5	$(AC)_{16}$	F: GGCTGAATGGCGTGTTTCC<br>R: CCCAACTGGTTCAACATCGG
Rhin_t_30	61.5	$(AG)_{13}$	F: ACTTGGATGGAAGGAGACCG<br>R: GGTCCTTTCTACGCTCACTG
Rhin_t_31	63.8	$(AC)_{14}$	F: GCAGCCAATCCCATCATACG<br>R: AAGCCCAGCATGTGTAATCC
Rhin_t_32	62.9	$(AC)_{18}$	F: AGGTTCTTAGAGGCTGGCAC<br>R: ACCAACATCCCAGGACTAGC
Rhin_t_46	61.3	$(AC)_{15}$	F: ACATCTTACCTTCCCACCTGG<br>R: GTGCATGTCTATCTGTCTTTGC
Rhin_t_47	62.4	$(AC)_{13}$	F: TCCCTGAAATCCTCTCAGCC<br>R: CTTCTGTGCTGTGTCTGCC

Supplementary Information 2: A: Structure diagram for K=2 (assumption of two populations). Even when multiple populations are assumed, the model resolves to a singular well mixed population as indicated by the dominance of grey (population 1) over yellow (population 2) within the plot. **B:** Evanno's deltaK results produced by STRUCTURE Harvester when the model was run for K=2 and K=3. This method cannot be applied to K=1 (the most likely scenario in the present case), but these results have been included for sake of completion and future comparison.

A

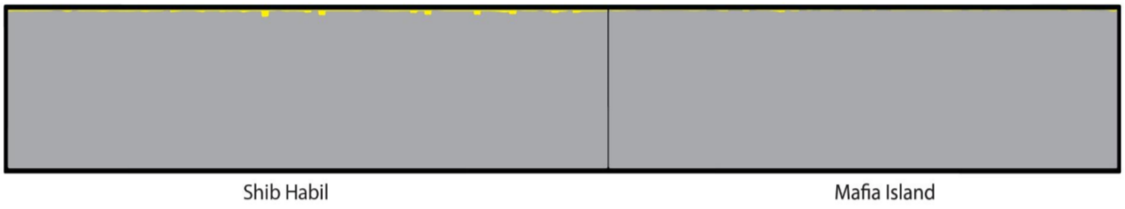

B

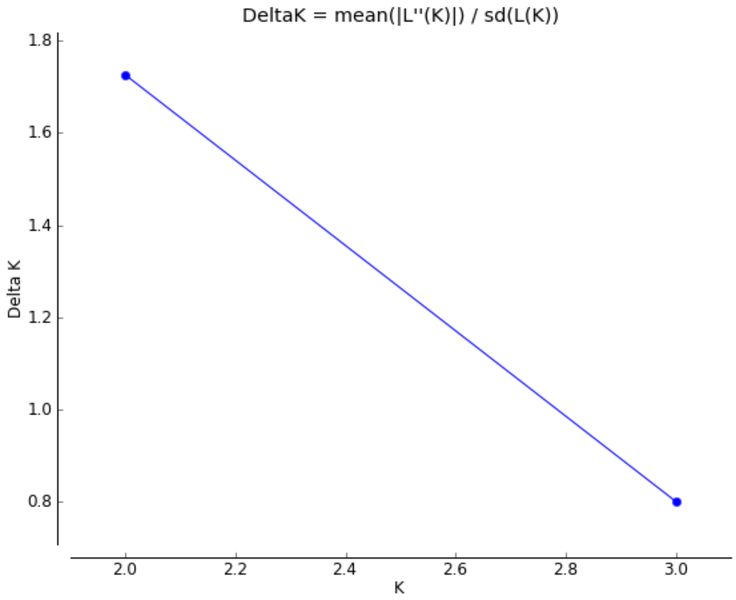

Supplementary Information 3: Previous studies from which whale shark mitochondrial control region sequences were sourced. Columns show number of sequences at locations used from each study in final alignment. Last column (light grey) is total number of sequences from each location used in final alignment, last row is overall sequence total. Bottom row (dark grey) is accession numbers if available associated with each previous publication. RS=Red Sea, Pac= Pacific, Atl= Atlantic.

	Castro *et al.* 2007	Ramírez-Macías *et al.* 2007	Schmidt *et al.* 2010	Vignaud et al. 2014	Sigsgaard *et al.* 2016	Meekan *et al.* 2017	Yagishita *et al.* 2020	This Study	**Totals**
**Saudi Arabia (RS)**	-	-	-	8	-	-	-	60	**68**
**Djibouti**	-	-	-	77	-	-	-	-	**77**
**Qatar**	-	-	-	-	54	-	-	-	**54**
**Tanzania**	-	-	-	-	-	-	-	57	**57**
**Mozambique**	18	-	-	31	-	13	-	-	**62**
**Seychelles**	-	-	-	31	-	7	-	-	**38**
**Maldives**	1	-	-	9	-	2	-	-	**12**
**W. Australia**	11	-	-	146	-	5	-	-	**162**
**Philippines**	11	-	-	20	-	-	-	-	**31**
**Taiwan**	-	-	1	25	-	-	-	-	**26**
**Japan**	-	-	-	-	-	-	28	-	**28**
**Mexico (Pac)**	8	36	-	77	-	-	-	-	**121**
**Mexico (Atl)**	19	-	-	61	-	-	-	-	**80**
Accession Numbers	EU182401-EU182444	DQ395261 – DQ395274	GU289922	n/a	KX944487 - KX944547	MF872682-MF872725	LC466742 – LC466769	OL782199-OL782316	**816**

Supplementary Information 4: Selection table for 24 candidate substitution models. Based on BIC values, the T29+G model using a gamma shape parameter of 0.48 was the most parsimonious and used to estimate genetic divergence in the mitochondrial data among sampling sites.

Model	BIC	ΔBIC	(+γ)
T92+G	28,074,443	0	0.48
GTR+G	28,083,985	9,542	0.45
T92+G+I	28,085,620	11,177	0.76
HKY+G	28,090,211	15,768	0.48
TN93+G	28,094,183	19,740	0.47
GTR+G+I	28,094,746	20,303	0.79
HKY+G+I	28,101,422	26,979	0.75
TN93+G+I	28,105,310	30,867	0.74
T92+I	28,115,638	41,195	-
GTR+I	28,130,459	56,016	-
HKY+I	28,134,289	59,846	-
TN93+I	28,137,737	63,294	-
K2+G	28,211,998	137,555	0.46
K2+G+I	28,223,730	149,287	0.66
JC+G	28,231,991	157,548	0.48
JC+G+I	28,243,833	169,390	0.68
K2+I	28,251,821	177,378	-
T92	28,257,219	182,776	-
HKY	28,276,322	201,879	-
JC+I	28,281,491	207,048	-
TN93	28,284,799	210,356	-
GTR	28,290,368	215,925	-
K2	28,392,085	317,642	-
JC	28,419,926	345,483	-

Supplementary Information 5: Relationships of *Rhincodon typus* haplotypes, from 13 different geographic locations (color in legend), in median-joining network created using whale shark mitochondrial control region sequences where gaps were **not** considered informative. Each circle represents a unique haplotype and is proportional to total haplotype frequency. Branches connecting circles represents a single nucleotide substitution; black crossbar represents an additional nucleotide substitution; black double slash bars represent more than 10 nucleotide substitutions (exact numbers noted). Areas encompassed by dashed lines represent four putative lineages. RS=Red Sea, Pac= Pacific, Atl= Atlantic.

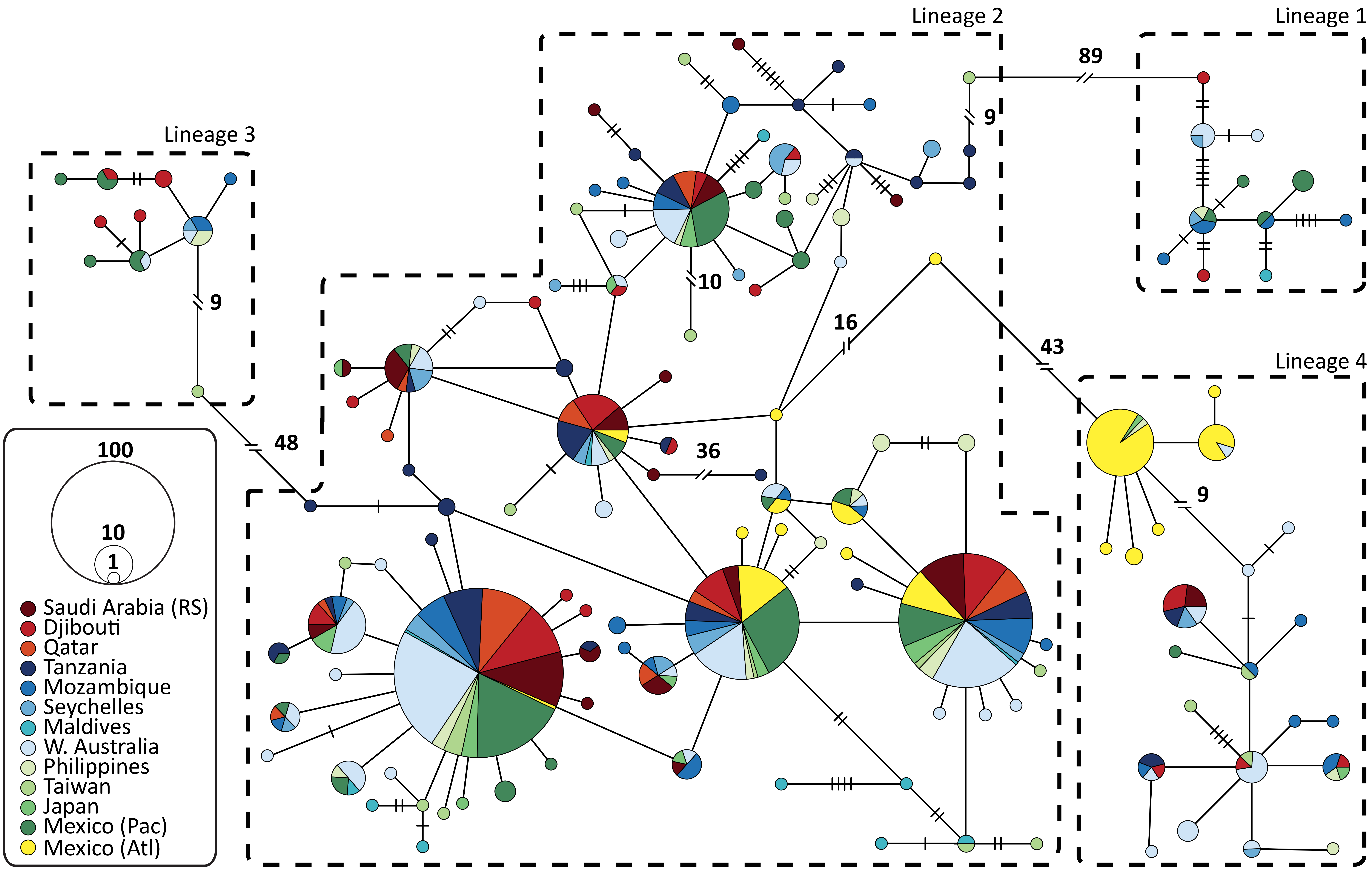
Lineage 1
Lineage 2
Lineage 3
Lineage 4
89
9
10
16
43
48
36
100
10
1
Saudi Arabia (RS)
Djibouti
Qatar
Tanzania
Mozambique
Seychelles
Maldives
W. Australia
Philippines
Taiwan
Japan
Mexico (Pac)
Mexico (Atl)

Supplementary Information 6: Population pairwise F_{st} and *P*-values based on haplotype frequencies, **A** gap-**exclusive** and **B** gap-**reduced** alignments. **-** Not significant; + P < 0.05; ++ P < 0.01 (corrected for multiple comparisons). RS=Red Sea, Pac= Pacific, Atl= Atlantic.

A

	Saudi Arabia (RS)	Djibouti	Qatar	Tanzania	Mozambique	Seychelles	Maldives	W. Australia	Philippines	Taiwan	Japan	Mexico (Pac)	Mexico (Atl)
**Saudi Arabia (RS)**		-	-	-	-	-	-	-	-	-	-	++	++
**Djibouti**	-0.0008		-	-	-	-	-	-	-	-	-	+	++
**Qatar**	-0.0069	0.0016		-	-	+	+	-	+	-	-	+	++
**Tanzania**	-0.0018	-0.006	-0.0014		-	-	-	-	-	-	-	-	++
**Mozambique**	0.0048	0.0036	0.0131	0.0056		-	-	-	-	-	-	+	++
**Seychelles**	0.0124	0.0051	0.023	0.0088	0.0088		-	-	-	-	-	-	++
**Maldives**	0.0311	0.0158	0.0449	0.0148	0.0088	0.0095		-	+	-	-	+	++
**W. Australia**	-0.0008	0.0008	0.0005	0.0025	0.0031	0.0118	0.0286			-	-	++	++
**Philippines**	0.008	0.0034	0.0199	0.004	-0.007	0.0028	-0.0042	0.0078		-	-	+	++
**Taiwan**	0.0069	0.0049	0.0067	0.0035	0.0019	0.007	0.0035	0.0021	0.0025		-	-	++
**Japan**	-0.0037	-0.0027	0.0064	0.0003	-0.0115	0.0104	0.0195	-0.0058	-0.0037	0.0078		-	++
**Mexico (Pac)**	0.0163	0.0124	0.0134	0.0082	0.0185	0.0179	0.0482	0.0099	0.021	0.0159	0.0091		++
**Mexico (Atl)**	0.1259	0.1052	0.1456	0.1116	0.0953	0.0955	0.0925	0.1153	0.0779	0.1136	0.0894	0.1182	

B

	Saudi Arabia (RS)	Djibouti	Qatar	Tanzania	Mozambique	Seychelles	Maldives	W. Australia	Philippines	Taiwan	Japan	Mexico (Pac)	Mexico (Atl)
**Saudi Arabia (RS)**		-	-	-	-	-	-	-	-	-	-	++	++
**Djibouti**	-0.0018		++	-	-	+	-	-	-	+	-	++	++
**Qatar**	0.0062	0.016		-	-	-	-	-	-	-	-	-	++
**Tanzania**	0.0011	0.0051	0.0016		-	-	-	-	-	-	-	+	++
**Mozambique**	0.0029	0.004	0.0067	0		-	-	-	-	-	-	-	++
**Seychelles**	0.0112	0.0109	0.0067	0.00446	0.0021		-	-	-	-	-	-	++
**Maldives**	0.0118	0.0063	0.0329	0.0067	0.0093	0.0165		-	-	-	-	-	++
**W. Australia**	0.0031	0.0074	0.0013	0.0063	0.0019	0.0062	0.025		-	-	-	+	++
**Philippines**	0.0029	0.0014	0.0075	-0.0026	-0.008	0	-0.0031	0.0044		-	-	+	++
**Taiwan**	0.0124	0.0176	0.0033	-0.0009	0.0055	0.0064	0.005	0.0091	0.0035		-	-	++
**Japan**	0.0025	-0.0007	0.0103	0.0073	-0.0094	0.0082	0.0071	0.0019	-0.0061	0.0164		-	++
**Mexico (Pac)**	0.0185	0.0189	0.0016	0.0101	0.0095	0.0103	0.0396	0.0072	0.0127	0.0108	0.0135		++
**Mexico (Atl)**	0.0913	0.08	0.109	0.0839	0.0746	0.0835	0.083	0.0899	0.0643	0.0991	0.0662	0.0982	

Supplementary Information 7: Measures of genetic diversity at each location (gaps **not** considered); number of sequences (n), number of haplotypes (N_{hp}), haplotype diversity (*h*), and nucleotide diversity (π), Tajima's *D*, Fu's *Fs*, and Harpending's raggedness index (HRI). NS Not significant; + 0.01< P < 0.05; ++ P < 0.01 (corrected for multiple comparisons). RS=Red Sea, Pac= Pacific, Atl= Atlantic.

**Sampling locations**	**Genetic diversity**				**Neutrality tests**		**Mismatch distribution (HRI)**
	**n**	N_{hp}	***h***	π	**Tajima's *D***	**Fu's *Fs***	
**Saudi Arabia (RS)**	68	18	0.86 ± 0.03	0.03 ± 0.02	-1.70^{+}	-0.24^{NS}	0.05^{NS}
**Djibouti**	77	26	0.89 ± 0.02	0.11 ± 0.06	-1.51^{+}	3.11^{NS}	0.02^{NS}
**Qatar**	54	15	0.83 ± 0.04	0.05 ± 0.03	-1.25^{NS}	2.67^{NS}	0.05^{NS}
**Tanzania**	57	21	0.89 ± 0.03	0.01 ± 0.01	-0.71^{NS}	-10.32^{++}	0.03^{NS}
**Mozambique**	62	28	0.93 ± 0.02	0.19 ± 0.09	-0.71^{NS}	3.50^{NS}	0.01^{NS}
**Seychelles**	38	17	0.93 ± 0.02	0.13 ± 0.06	-1.79^{+}	3.85^{NS}	0.04^{NS}
**Maldives**	12	12	1.00 ± 0.03	0.14 ± 0.08	-1.97^{++}	-2.44^{NS}	0.05^{NS}
**W. Australia**	162	40	0.87 ± 0.02	0.09 ± 0.04	-1.56^{+}	0.06^{NS}	0.02^{NS}
**Philippines**	31	18	0.95 ± 0.02	0.13 ± 0.07	-1.85^{++}	1.68^{NS}	0.01^{NS}
**Taiwan**	26	19	0.93 ± 0.04	0.09 ± 0.05	-1.37^{NS}	-1.54^{NS}	0.01^{NS}
**Japan**	28	12	0.90 ± 0.03	0.05 ± 0.02	-1.52^{+}	-1.19^{NS}	0.03^{NS}
**Mexico (Pac)**	121	25	0.85 ± 0.02	0.10 ± 0.05	-1.61^{+}	5.28^{NS}	0.02^{NS}
**Mexico (Atl)**	80	16	0.81 ± 0.03	0.13 ± 0.06	3.62^{NS}	17.41^{NS}	0.11^{NS}
**Indo-Pacific**	736	178	0.89 ± 0.01	0.09 ± 0.05	-1.45^{+}	-23.41^{+}	0.02^{NS}
**Overall**	**816**	**192**	**0.90 ± 0.01**	**0.11 ± 0.05**	**-1.18^{NS}**	**-23.32^{+}**	**0.01^{NS}**

Supplementary Information 8: Neutrality statistics at each location (gap-**reduced**); number of sequences (n), Tajima's *D*, Fu's *Fs*, and Harpending's raggedness index (HRI). NS Not significant; + 0.01< P < 0.05; ++ P < 0.01 (corrected for multiple comparisons). RS=Red Sea, Pac= Pacific, Atl= Atlantic.

**Sampling locations**	**Neutrality tests**			**Mismatch distribution (HRI)**
	**n**	**Tajima's *D***	**Fu's *Fs***	
**Saudi Arabia (RS)**	68	-1.65^{+}	1.38^{NS}	0.03^{NS}
**Djibouti**	77	-1.39^{NS}	6.71^{NS}	0.02^{NS}
**Qatar**	54	-1.22^{NS}	5.25^{NS}	0.02^{NS}
**Tanzania**	57	-0.56^{NS}	-3.67^{NS}	0.01^{NS}
**Mozambique**	62	-0.60^{NS}	6.25^{NS}	0.01^{NS}
**Seychelles**	38	-1.66^{+}	5.32^{NS}	0.02^{+}
**Maldives**	12	-1.94^{++}	-1.45^{NS}	0.06^{NS}
**W. Australia**	162	-1.44^{+}	4.84^{NS}	0.02^{+}
**Philippines**	31	-1.73^{+}	2.04^{NS}	0.01^{NS}
**Taiwan**	26	-1.36^{NS}	-0.83^{NS}	0.01^{NS}
**Japan**	28	-1.41^{NS}	2.83^{NS}	0.02^{NS}
**Mexico (Pac)**	121	-1.51^{+}	12.50^{NS}	0.01^{NS}
**Mexico (Atl)**	80	3.70^{NS}	23.88^{NS}	N/A
**Indo-Pacific**	736	-1.32^{NS}	-23.10^{+}	0.01^{NS}
**Overall**	**816**	$\mathbf{-1.04^{NS}}$	$\mathbf{-21.67^{NS}}$	**N/A**

Supplementary Information 9: Gap-inclusive (left), gap-reduced (center), and gap-exclusive (right) Bayesian skyline plots for the overall dataset (top), Indo-Pacific (center), and Atlantic. The plots compare estimated population sizes (effective population times the mutation rate: $N_e\mu$) to time in substitutions per site. The bounding lines represent the 95% highest posterior density.

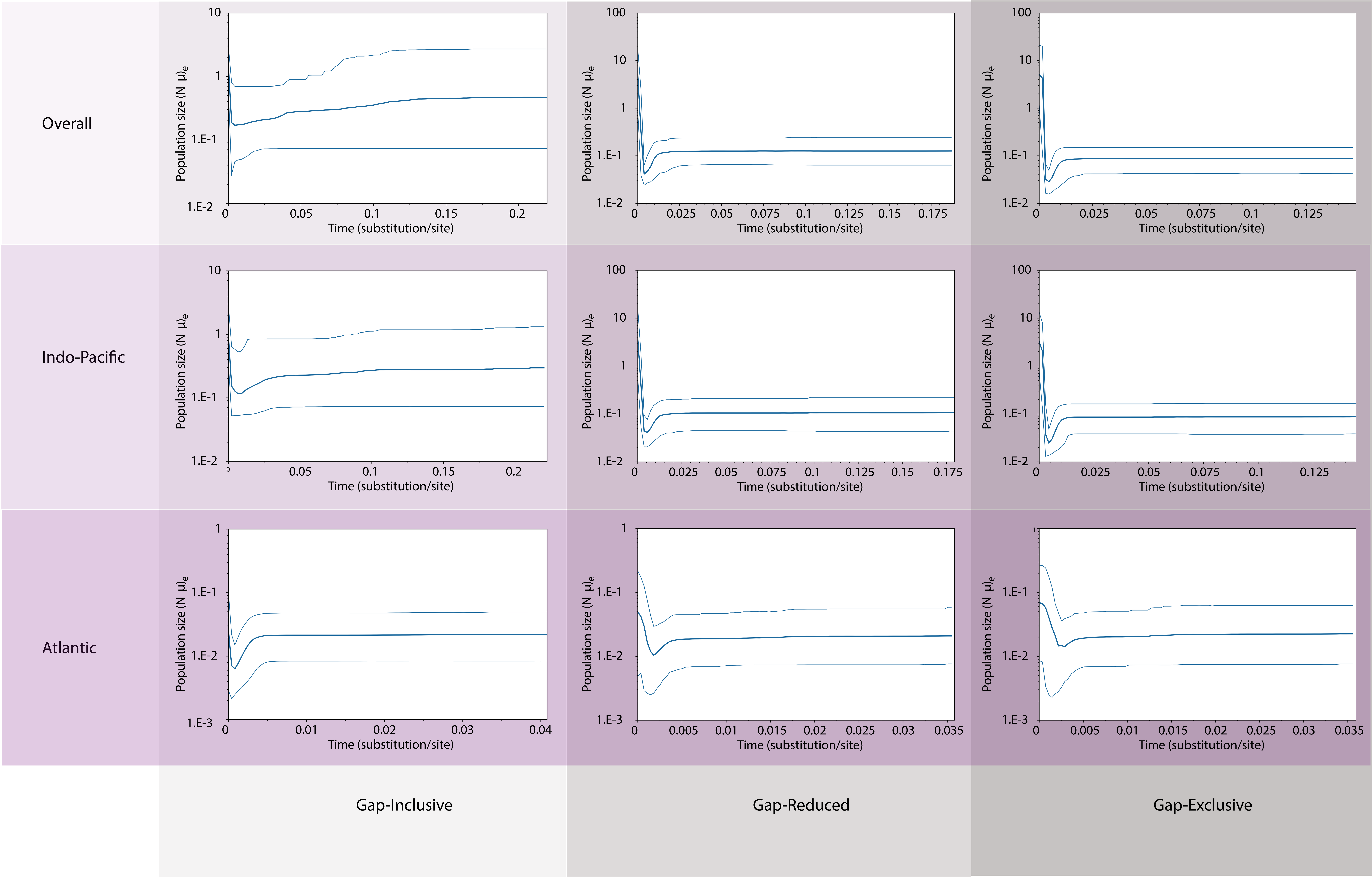
Overall
Indo-Pacific
Atlantic
Gap-Inclusive
Gap-Reduced
Gap-Exclusive
Population size (N μ)e
Time (substitution/site)
100
10
1
1.E-1
1.E-2
1.E-3
0
0.05
0.1
0.15
0.2
0.025
0.075
0.125
0.175
0.01
0.02
0.03
0.04
0.005
0.015
0.035

Supplementary Information 10: Metadata associated with all individuals sampled in the Red Sea, including Wildbook ID and accession number. *Sequences from Vignaud et al. 2014 that have been updated for present study **haplotypes from alignment where gaps were considered informative.

Wildbook	Sequence name	Sex	Size	Publications	Accession	Haplotype**	Lineage
R-167	RS16_RedSea	M	5	Vignaud	-	Hap 1	L2
R-042	RS24_RedSea	F	3	Vignaud	-	Hap 2	L2
R-171	RS26_RedSea	F	6	Vignaud	-	Hap 3	L2
R-038	RS38_RedSea	F	3	Vignaud	-	Hap 4	L4
R-039	RS44_RedSea	F	4	Vignaud	-	Hap 4	L4
R-086	RS46_RedSea	M	3	Vignaud	-	Hap 4	L4
R-072	RS49_RedSea	F	3.5	Vignaud	-	Hap 5	L2
R-094	RS53_RedSea	F	3	Vignaud	-	Hap 7	L2
-	UK3_RedSea	M	-	-	OL782199	Hap 8	L2
-	UK4_RedSea	-	-	-	OL782200	Hap 9	L2
R-061	UK6_RedSea	F	4.5	-	OL782201	Hap 10	L2
R-091	UT014_RedSea	F	4.5	-	OL782202	Hap 11	L2
R-088	UT015_RedSea	F	4	Vignaud*	OL782203	Hap 6	L2
R-109	UT104_RedSea	M	3.5	-	OL782204	Hap 12	L2
R-191	UT201_RedSea	F	5	-	OL782205	Hap 13	L2
R-127	UT301_RedSea	F	-	-	OL782206	Hap 14	L2
R-128	UT303_RedSea	F	-	-	OL782207	Hap 15	L2
R-159	UT304_RedSea	M	-	-	OL782208	Hap 6	L2
R-133	UT305_RedSea	F	-	-	OL782209	Hap 6	L2
R-157	UT307_RedSea	F	-	-	OL782210	Hap 16	L2
R-158	UT308_RedSea	F	-	-	OL782211	Hap 17	L2
R-147	UT503_RedSea	F	4.5	-	OL782212	Hap 6	L2
R-148	UT507_RedSea	-	-	-	OL782213	Hap 18	L2
R-151	UT512_RedSea	M	3.5	-	OL782214	Hap 19	L2
R-069	UT514_RedSea	F	-	-	OL782215	Hap 6	L2
R-165	WS003_RedSea	-	4.5	Vignaud*	OL782216	Hap 2	L2
R-163	WS005_RedSea	-	4	Vignaud*	OL782217	Hap 1	L2
R-035	WS008_RedSea	F	3	Vignaud*	OL782218	Hap 20	L2
R-031	WS009_RedSea	F	5	-	OL782219	Hap 2	L2
R-036	WS014_RedSea	M	3.5	Vignaud*	OL782220	Hap 2	L2
R-084	WS020_RedSea	F	4	-	OL782221	Hap 1	L2
R-169	WS023_RedSea	M	6.5	Vignaud*	OL782222	Hap 17	L2
R-030	WS028_RedSea	M	4	-	OL782223	Hap 21	L2
R-172	WS029_RedSea	F	7	Vignaud*	OL782224	Hap 22	L2
R-177	WS030_RedSea	M	3	Vignaud*	OL782225	Hap 2	L2
R-037	WS031_RedSea	M	3.5	Vignaud*	OL782226	Hap 23	L2
R-040	WS032_RedSea	M	-	Vignaud*	OL782227	Hap 1	L2
R-058	WS033_RedSea	F	4	Vignaud*	OL782228	Hap 6	L2
R-173	WS037_RedSea	M	7	Vignaud*	OL782229	Hap 6	L2
R-176	WS041_RedSea	F	4	Vignaud*	OL782230	Hap 24	L2
R-090	WS042_RedSea	M	3	Vignaud*	OL782231	Hap 25	L2
R-096	WS043_RedSea	F	3.5	Vignaud*	OL782232	Hap 24	L2

R-099	WS045_RedSea	M	3.5	-	OL782233	Hap 26	L2
R-070	WS103_RedSea	M	4	Vignaud*	OL782235	Hap 6	L2
R-085	WS104_RedSea	F	3	-	OL782236	Hap 18	L2
R-078	WS105_RedSea	M	-	-	OL782237	Hap 6	L2
R-073	WS107_RedSea	F	4	-	OL782238	Hap 27	L2
R-181	WS108_RedSea	F	4.5	-	OL782239	Hap 1	L2
R-182	WS109_RedSea	F	4	-	OL782240	Hap 2	L2
R-183	WS110_RedSea	F	4.5	-	OL782241	Hap 20	L2
R-098	WS112_RedSea	M	5	-	OL782242	Hap 2	L2
R-186	WS114_RedSea	F	3.5	-	OL782243	Hap 1	L2
R-199	WS118_RedSea	-	3	-	OL782244	Hap 6	L2
R-188	WS120_RedSea	F	3.5	-	OL782245	Hap 28	L2
-	WS123_RedSea	M	3.5	-	OL782246	Hap 1	L2
R-092	WS125_RedSea	F	4	-	OL782247	Hap 15	L2
R-190	WS126_RedSea	M	3	-	OL782248	Hap 2	L2
R-198	WS203_RedSea	M	3	-	OL782249	Hap 20	L2
R-134	WS206_RedSea	M	3.5	-	OL782250	Hap 2	L2
R-201	WS207_RedSea	F	3	-	OL782251	Hap 15	L2
R-200	WS208_RedSea	F	4	-	OL782252	Hap 2	L2
R-202	WS211_RedSea	M	4.5	-	OL782253	Hap 21	L2
R-189	WS212_RedSea	F	4	-	OL782254	Hap 6	L2
R-142	WS503_RedSea	M	5	-	OL782255	Hap 29	L2
R-141	WS504_RedSea	M	4.5	-	OL782256	Hap 6	L2
R-059	WS508_RedSea	M	3.5	-	OL782257	Hap 30	L2
R-105	WS509_RedSea	-	-	-	OL782258	Hap 17	L2
R-106	WS510_RedSea	M	3.5	-	OL782259	Hap 31	L2

Supplementary Information 11: Metadata associated with all individuals sampled at Mafia Island, Tanzania, including Wildbook ID and accession number. **Haplotypes from alignment where gaps were considered informative.

Wildbook	Sequence name	Sex	Size	Accession	Haplotype**	Lineage
-	M104_Tanzania	-	-	OL782260	Hap 15	L2
-	MF10_Tanzania	-	-	OL782261	Hap 12	L2
-	MF39_Tanzania	M	6	OL782262	Hap 1	L2
TZ-068	MF62_Tanzania	F	6	OL782263	Hap 65	L2
MZ-129	MZ129_Tanzania	M	6	OL782264	Hap 66	L2
TZ-001	TZ001_Tanzania	M	8	OL782265	Hap 67	L2
TZ-003	TZ003_Tanzania	M	8	OL782266	Hap 2	L2
TZ-009	TZ009_Tanzania	M	6.5	OL782267	Hap 20	L2
TZ-010	TZ010_Tanzania	M	8	OL782268	Hap 3	L2
TZ-011	TZ011_Tanzania	M	7	OL782269	Hap 6	L2
TZ-014	TZ014_Tanzania	M	6	OL782270	Hap 19	L2
TZ-016	TZ016_Tanzania	M	7	OL782271	Hap 68	L2
TZ-017	TZ017_Tanzania	M	7	OL782272	Hap 3	L2
TZ-019	TZ019_Tanzania	M	6	OL782273	Hap 69	L2
TZ-020	TZ020_Tanzania	M	6	OL782274	Hap 70	L2
TZ-023	TZ023_Tanzania	M	5	OL782275	Hap 2	L2
TZ-025	TZ025_Tanzania	M	7	OL782276	Hap 71	L2
TZ-026	TZ026_Tanzania	M	6	OL782277	Hap 18	L2
TZ-027	TZ027_Tanzania	M	7.5	OL782278	Hap 6	L2
TZ-028	TZ028_Tanzania	M	6	OL782279	Hap 72	L2
TZ-029	TZ029_Tanzania	M	7	OL782280	Hap 2	L2
TZ-030	TZ030_Tanzania	M	4.5	OL782281	Hap 2	L2
TZ-032	TZ032_Tanzania	M	5	OL782282	Hap 73	L2
TZ-035	TZ035_Tanzania	M	6	OL782283	Hap 3	L2
TZ-036	TZ036_Tanzania	F	5	OL782284	Hap 74	L2
TZ-038	TZ038_Tanzania	M	5	OL782285	Hap 2	L2
TZ-040	TZ040_Tanzania	M	4.5	OL782286	Hap 2	L2
TZ-042	TZ042_Tanzania	M	8	OL782287	Hap 54	L2
TZ-043	TZ043_Tanzania	M	5	OL782288	Hap 75	L2
TZ-044	TZ044_Tanzania	M	6	OL782289	Hap 76	L2
TZ-045	TZ045_Tanzania	M	5	OL782290	Hap 20	L2
TZ-046	TZ046_Tanzania	M	6	OL782291	Hap 1	L2
TZ-048	TZ048_Tanzania	M	5	OL782292	Hap 20	L2
TZ-054	TZ054_Tanzania	F	6	OL782293	Hap 1	L2
TZ-055	TZ055_Tanzania	F	5	OL782294	Hap 1	L2
TZ-056	TZ056_Tanzania	M	5	OL782295	Hap 15	L2
TZ-057	TZ057_Tanzania	M	5	OL782296	Hap 21	L2
TZ-059	TZ059_Tanzania	M	4	OL782297	Hap 6	L2
TZ-060	TZ060_Tanzania	M	8	OL782298	Hap 2	L2
TZ-061	TZ061_Tanzania	M	4	OL782299	Hap 10	L2
TZ-064	TZ064_Tanzania	M	7	OL782300	Hap 77	L2
TZ-065	TZ065_Tanzania	M	7	OL782301	Hap 6	L2

TZ-066	TZ066_Tanzania	F	6	OL782302	Hap 6	L2
TZ-067	TZ067_Tanzania	M	5	OL782303	Hap 6	L2
TZ-070	TZ070_Tanzania	M	5	OL782304	Hap 78	L2
TZ-074	TZ074_Tanzania	M	6	OL782305	Hap 79	L2
TZ-079	TZ079_Tanzania	M	6	OL782306	Hap 54	L2
TZ-083	TZ083_Tanzania	F	6	OL782307	Hap 21	L2
TZ-084	TZ084_Tanzania	F	5	OL782308	Hap 80	L2
TZ-085	TZ085_Tanzania	M	5	OL782309	Hap 81	L2
TZ-086	TZ086_Tanzania	M	7	OL782310	Hap 82	L2
TZ-088	TZ088_Tanzania	M	5.5	OL782311	Hap 3	L2
TZ-089	TZ089_Tanzania	M	4.5	OL782312	Hap 83	L2
TZ-090	TZ090_Tanzania	M	7	OL782313	Hap 2	L2
TZ-092	TZ092_Tanzania	M	6	OL782314	Hap 2	L2
TZ-093	TZ093_Tanzania	M	3.5	OL782315	Hap 84	L2
TZ-096	TZ096_Tanzania	M	6	OL782316	Hap 85	L2
